# Could Fostering Alternative Plant Feedstocks Improve the Sustainability of Leather Manufacturing? A Critical Review

**DOI:** 10.3390/ma18163759

**Published:** 2025-08-11

**Authors:** Valentina Beghetto, Vanessa Gatto, Silvia Conca

**Affiliations:** 1 Crossing S.r.l., Viale della Repubblica 193/b, 31100 Treviso, Italy; vanessa.gatto@crossing-srl.com (V.G.); silvia.conca@crossing-srl.com (S.C.); 2 Department of Molecular Sciences and Nanosystems, University Ca’ Foscari of Venice, Via Torino 155, 30172 Mestre, Italy; 3 Consorzio Interuniversitario per le Reattività Chimiche e La Catalisi (CIRCC), Via C. Ulpiani 27, 70126 Bari, Italy

**Keywords:** vegetable tannins, alternative tanning, sustainable leather production, green tanning

## Abstract

Vegetable tannins (VTs) are natural polyphenolic compounds widely used in leather tanning as sustainable alternatives to chrome-based processes. Traditionally, only a limited number of commercially available tannins, such as mimosa, quebracho, and chestnut, are employed globally, often requiring long-distance transportation with associated environmental and economic costs. This review systematically explores recent advances (2015–2025) in the identification and evaluation of alternative VT sources derived from underutilized plant species in Africa and Asia. Chemical composition, extraction efficiency, and tanning performance, including hydrothermal stability, tensile strength (TS), elongation at break (EB%), and tear strength (Ts), are critically analyzed and compared with conventional agents. Particular focus is given to the tannin/non-tannin ratio (T/N), a key indicator of tanning potential. Promising results were found for extracts from *Acacia xanthophloea*, *Cassia singueana*, *Solanum incanum*, *Pontederia crassipes*, and *Xylocarpus granatum*. Preliminary environmental assessments (COD, BOD, TDS) also suggest comparable impacts to standard tannins. However, performance variability due to species, plant part, seasonality, and extraction conditions remains a challenge. This review underscores the potential of regionally sourced VTs to support proximity-based economies and reduce the environmental footprint of the leather industry, while highlighting the need for further studies to optimize extraction protocols and scale industrial application.

## 1. Introduction

**Figure 1:**
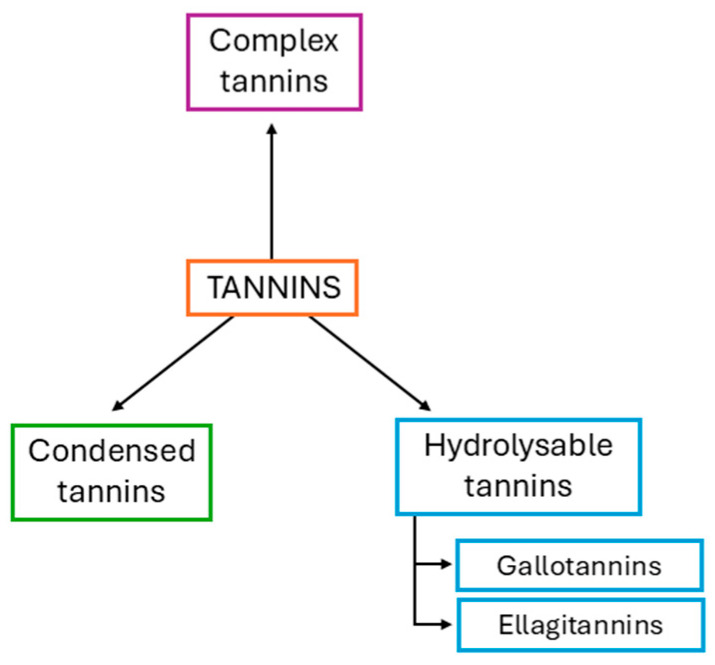
Tannin classification.

The industrial history of the tannin industry started in the late 18th century, and the word ‘tannin’ refers to vegetable extracts used to transform hides into leather [1,2]. Vegetable tannins (VTs) are naturally occurring water-soluble polyphenolic compounds present in tree barks, stems, leaves, seeds, roots, and in different nuts, fruits, spices, and herbs. They have a molecular weight between 500 and 30,000 Da and are classified into hydrolysable tannins, condensed tannins, and complex tannins (Figure 1) [3,4].

**Figure 2:**
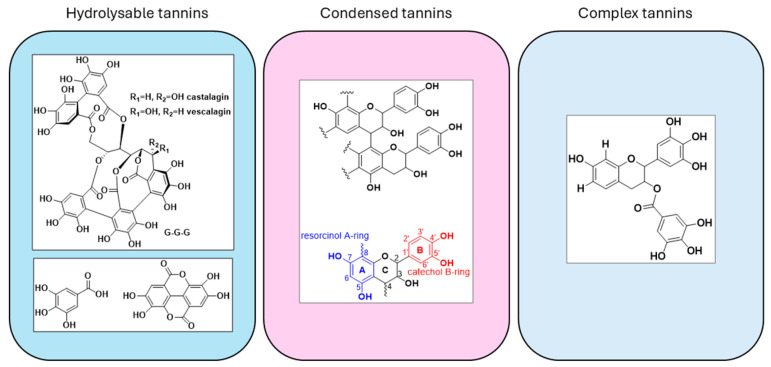
Structures of hydrolysable tannins, condensed tannins and complex tannins.

Hydrolysable tannins are present in variable amounts in different plants and are formed by a carbohydrate core (glucose), esterified with gallic acid (gallotannins) or ellagic acid (ellagitannins) (Figure 2). The name derives from the fact that they are easily hydrolysed by acids or bases [5]. Condensed tannins (or proanthocyanidins) consist of flavonoid units (mainly flavan-3-ols) connected through C4–C6 or C4–C8 bonds (Figure 2), while complex tannins are a combination of hydrolysable and condensed tannins such as, for example, Acutissimim A, which is composed of an ellagitannin or gallotannin combined with a flavan-3-ol unit (Figure 2) [3,6].

Tannins are widely used at the industrial level [7] for food and beverages [6,8], pharmaceuticals [8,9], leather tanning [10,11,12,13,14], adhesives [15,16], dyes [17], cosmetics [18,19] and animal nutrition [20,21].

**Figure 3:**
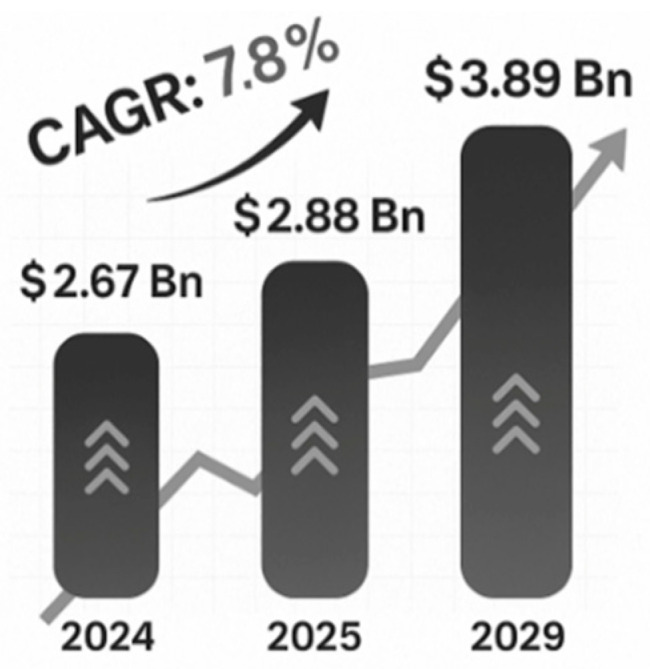
Global tannin market.

The global tannin market was valued at USD 2.67 billion in 2024 and is expected to expand at a compound annual growth rate (CAGR) of 7.8% from 2023 to 2029 (Figure 3). Leather tanning is emerging as the largest application segment, accounting for almost 62% of the total market revenues in 2022, 90% of which is contributed by condensed tannins [22].

Known since prehistoric times for their ability to transform putrescible animal hides into stable materials [5,23], VTs remain a valuable solution for the tanning of hides as alternatives to chrome salts [5], aldehydes [24,25] and synthetic tannins [26,27,28].

Presently, over 85% of leather products are tanned with Cr(III) salts (‘wet blue’) due to their superior quality, cost-effectiveness, versatility, and replicability. Nevertheless, this reliance comes at a significant environmental cost, producing large volumes of chromium-containing solid sludge, which leads to high disposal expenses and considerable ecological burden. Consequently, these environmental issues, combined with stringent European regulations, are gradually accelerating the adoption of novel, sustainable and chrome-free tanning systems for leather manufacturing [10,28,29,30]. The most recent literature regarding the use of VTs has been devoted to their use in combination metal tanning systems [31,32], for the synthesis of biobased syntans [28,33] and for the implementation of new high-exhaustion systems for tannery wastewater purification [32,34,35,36]. Another interesting research area which has gained increasing interest in recent years is the study of the tanning efficiency of new vegetable feedstocks present in different geographical areas around the world, which may both increase their availability and promote virtuous proximity economy business models [37,38,39]. In fact, proximity economy consists of local and short-value chains, reducing transportation costs and environmental impact, promoting local production and consumption and economic and innovative performance, and stimulating sustainable regional development and circular economy [40,41]. The proximity of tannin industrial plants to tanneries constitutes an important reduction in transport and therefore in the CO_2_ debts generated by the handling of large quantities of raw materials, with a consequent reduction in costs and benefit to the life cycle of the finished leather products. Wijeyekoon and coworkers, for example, reported a study on symbiotic benefits deriving from the co-production of bark briquettes and tannins from wood bark in New Zealand, an important player in the leather industry market, reducing new investment costs and significantly improving the socio-economic and environmental sustainability of both processes [42].

The leather industry has traditionally relied on a limited selection of imported VTs, but to address the growing demand for tanning agents and leather goods worldwide, it is essential to explore new, locally available alternatives. Additionally, within a single plant species, tannin distribution varies in the different parts of the plant (bark, wood, leaves) [43] and plant extracts contain both tannin and non-tannin compounds [44]. Therefore, the possibility of exploiting new vegetable sources for the production of tannins requires careful analysis and evaluation. Additionally, if these new extracts need to be used for leather tanning, particular attention should be given to the tannin over non-tannin ratio (T/N). In fact, tannins play a crucial role in tanning and retanning steps, influencing the mechanical properties of the finished leather, such as tensile strength, Ts, and crack resistance [45]. On the other hand, non-tannin substances, which are loosely associated with collagen (acids, salts, sugars, low-molecular-weight polyphenols and polysaccharides), do not contribute to the tanning of leather but generally influence colour and texture and impact the quality of wastewater [23,29].

In this context, the scope of this work is to provide an overview of the state of the art regarding the identification of alternative vegetable feedstocks available in different geographical areas, as well as their characterization and efficiency as pre-tanning, tanning and retanning agents in leather processing. The chemical composition of the different extracts and physical mechanical characteristics of leather produced will be given and compared to conventional tanning systems where possible. Additionally, impacts such as chemical oxygen demand (COD), biological oxygen demand (BOD) and other environmental data will be reported and discussed, when available. The technology readiness level and commercial availability of these alternative sources can differ substantially. In fact, many alternative tannins are currently in the research and development phase, so extensive investigation into extraction standardization, tanning process optimization, and production scalability is required.

To the best of our knowledge, although many reviews have been reported on the use of commercially available VTs [4,11,12,23], this is the first review on the valorisation of alternative VTs from different geographical areas, for the leather industry.

## 2. Methodology

A systematic search of the existing literature was conducted to thoroughly analyze the current status of new alternative feedstocks for the extraction of tannins for the leather industry, giving a critical evaluation and enabling new perspectives for knowledge enhancement. A comprehensive analysis of the literature regarding leather tanning was mapped using a combination of different keywords such as “vegetable tannins”, “alternative vegetable tanning”, “vegetable leather”, “tannins feedstock”, “geographical distribution of vegetable tannins for leather”, “alternative vegetable tannins for leather”, “sustainable tanning”, “improved tanning”, “alternative tanning”, “condensed tannins”, “hydrolysable tannins”, “wet-white tanning”, “sustainable leather production”, and “green tanning”. Specifically, one or a combination of two keywords were chosen for the selection of the papers and only those published in the English language in peer-reviewed journals have been reported.

Research papers included in the review were collected through Web of Science, Scopus, Google Scholar, ScienceDirect, and ResearchGate, and were published between 2015 and 2025. Only papers containing both VTs extraction procedures from new cultivars and tanning tests have been reported. The last search was performed on May 30, 2025. The search allowed us to identify 15 relevant papers, which are described below.

## 3. Geographical Distribution, Market and Extraction of Newly Sourced Vegetable Tannins

### 3.1. Geographical Distribution and Market of Most Commonly Employed VTs

**Figure 4:**
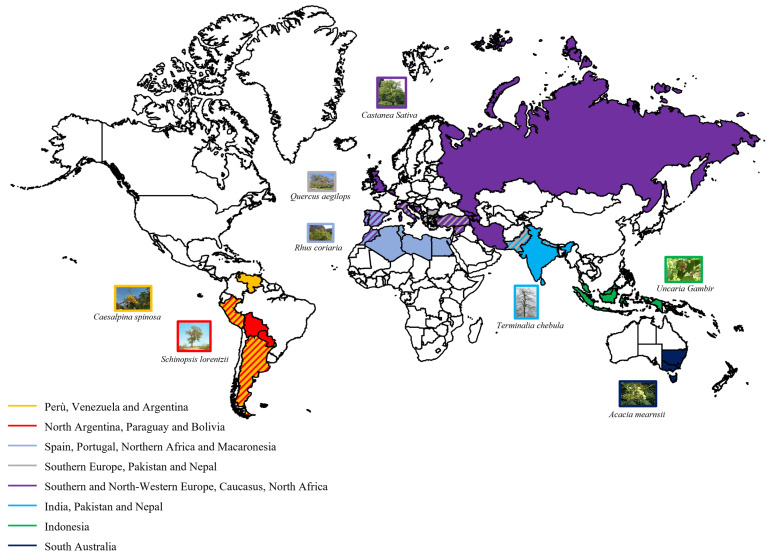
Geographical origin of most employed hydrolysable and condensed tannins, evidenced in different colours.

Most available VTs employed today by the leather industry are mimosa (*Acacia mearnsii*), quebracho (*Schinopsis lorentzii*), and gambir (*Uncaria gambir*) (condensed tannins) and to lesser extent chestnut (*Castanea sativa*), myrobalan (*Terminalia chebula*), valonia (*Quercus aegilops*), tara (*Caesalpinia Spinosa*), and sumac (*Rhus coriaria*) (hydrolysable tannins). They grow in specific geographical areas (Figure 4). Condensed tannins mainly come from South Australia (*Acacia mearnsii*), North Argentina, Paraguay and Bolivia (*Schinopsis lorentzii*), and Indonesia (*Uncaria gambir*), while hydrolysable tannins are mainly produced from plants originating from Southern and North-Western Europe, the Caucasus, North Africa (*Castanea sativa*), India (*Terminalia chebula*), Pakistan, Nepal and Southern Europe (*Quercus aegilops*), Perú, Venezuela and Argentina (*Caesalpinia Spinosa*) and Macaronesia (group of islands in the Atlantic Ocean) (*Rhus coriaria*) (Figure 4) [11,12,23,46,47,48].

The limited geographical distribution of most commercially available VTs implies that most countries producing leather need to import extracts from distant locations, with considerable environmental impacts deriving from transportation. According to Conde and coworkers, maritime transport from Eastern Africa and South America to Europe is the second most impactful stage in terms of CO_2_ emissions for mimosa extraction [49]. Their research further highlights the significant environmental benefit of local sourcing: the adoption of local VT feedstocks, such as pine bark compared with imported mimosa, has been shown to result in over 90% CO_2_-equivalent savings, primarily by eliminating long-distance transport emissions. Thus, the exploitation of other natural resources as feedstocks for VTs, to be used for the tanning of hides, close to leather production plants, reduces transportation costs and impacts, contributing to improving the environmental sustainability of leather goods. Additionally, less developed countries will greatly benefit from the exploitation of autochthonous species, since imported VTs are economically unsustainable, pushing these countries to adopt cheap chrome salts instead of more environmentally friendly solutions such as VTs.

### 3.2. Geographical Distribution and Market of Newly Sourced VTs

**Figure 5:**
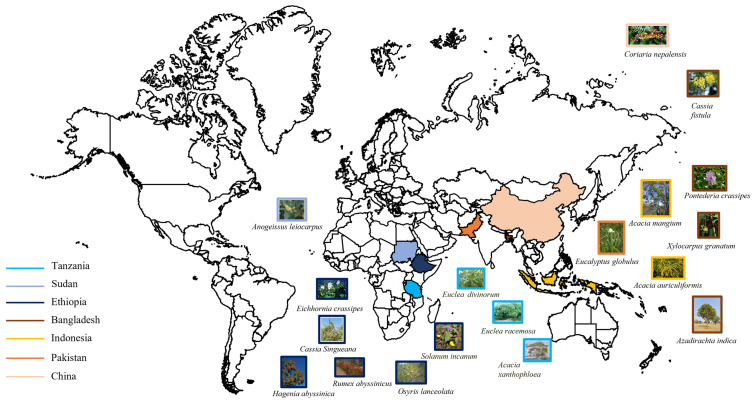
Geographical origin of novel VTs.

Based on the literature analysis performed as reported in Section 2, various new sources of VTs from different geographical areas have been reported [37,45,50,51,52,53,54,55,56,57,58,59,60,61,62] (Figure 5). In Africa, condensed tannins species have been found, especially in Tanzania (*Acacia xanthophloea*, *Euclea divinorum* and *Euclea racemosa*), Sudan (*Anogesus leiocarpus*) and Ethiopia (*Cassia singueana*, *Solanum incanum*, *Rumex abyssinicus*, *Eichhornia crassipes*, *Hagenia abyssinica* and *Osyris lanceolata*). South Asia also has some new sources of condensed tannins, in Bangladesh (*Cassia fistula*, *Pontederia crassipes*, *Xylocarpus granatum*, *Azadirachta indica*), in Indonesia (*Acacia mangium* and *Acacia auriculiformis*) and in Pakistan (*Eucalyptus globulus*). Only *Coriaria Nepalensis* from Nepal contains hydrolysable tannins (Table 1).

In this paper, according to the geographical origin of the new condensed and hydrolysable tannin studied, different sections have been dedicated to describing the extraction protocols and their characterization, followed by their application and efficiency as tanning agents.

A summary of the different plants and new extracts reviewed in this work is reported in Table 1. The most important parameters, such as tannin/non-tannin ratio (T/N), tannin percentage (T(%)), total phenol content (TPC(%)), total flavonoid content (TFC(%)), total extraction yield (%), ash percentage (Ash %) and pH of the extracts, are given in Table 1.

From an industrial point of view, when a new extract is evaluated, one of the most important parameters measured in determining its potential commercial application is the tannin/non-tannin ratio (T/N), which should be at least >1 and preferably >2. In fact, the non-tannin fraction has no tanning efficiency, reduces overall extraction yields and negatively contributes to an increment in chemical oxygen demand (COD) and biological oxygen demand (BOD) of wastewater [50,63,64,65]. Additionally, it has recently been demonstrated that the use of purified chestnut and quebracho tannins, which contain very low non-tannin content (T/N of 8.3 and 11.2, respectively), not only improves the quality of wastewater but also produces bright white tanned hides, overcoming one of the important drawbacks of VTs, which usually generate yellowish leather with low light stability [29]. The T/N ratio may vary considerably not only with vegetable feedstock, geographical origin and seasonality, but also due to extraction conditions [66].

Also, tannin percentage (T(%)), total phenol content (TPC(%)) and total flavonoid content (TFC(%)) are critical factors that influence the quality and properties of leather. Higher tannin content, for example, generally leads to better leather durability and strength, while lower tannin content may result in softer, more flexible leather. Different plant sources have varying tannin contents. For example, quebracho wood can contain 14–26% tannin in its heartwood and 22–45% in its bark, while some bark extracts may have much higher tannin content [12]. Analogously, TPC (%) heavily depends on the source of the extract, and in particular for condensed tannins (quebracho extracts) the TFC (%) is very high due to the high concentration of proanthocyanidins [67,68].

Another important parameter that demonstrates the potential of an extract for leather tanning is the pH; the required pH range for effective leather tanning is between 4 and 6 [5,69].

Condensed and hydrolysable tannins are reported below in separate sections, according to their geographical origin.

### 3.3. Condensed Tannin Extracts

#### 3.3.1. AFRICA-Tanzania: *A. xanthopea*, *E. divinorum* and *E. racemosa* Extracts

China and coworkers recently reported a systematic study on the extraction and tanning efficiency of three different extracts, *Acacia xanthophloea*, *Euclea divinorum* and *Euclea racemosa*, which are easily available in Tanzania and traditionally employed by local cottage tanneries [50]. Taking inspiration from an old Tanzanian practice, the authors systematically studied different extraction conditions, cultivar origins and compositions of the extracts in order to define a protocol for their characterization and use for leather tanning in Tanzania, implementing socio-economically and environmentally benign practices. Extractions were carried out at varying temperatures from 30 to 80 °C for 4 h. Interestingly, an extract with a very high T/N ratio (6.6) was obtained, at 50 °C, from *A. xanthophloea*, and was produced in commercially acceptable quantities due to its yield, as well as T(%), TPC% and TFC%, as compared to commercially available *Acacia mearnsii* (Table 1).

A TNBSA (2,4,6-trinitrobenzenesulphonic acid) hydrolysis assay was used to determine the interaction mechanism between collagen and tannins and assess whether *A. xanthophloea*, *E. divinorum*, and *E. racemosa* contain condensed tannins [70]. It is important to note, however, that the extract from *E. racemosa* contains a low tannin content, as reported in Table 1, and gave unsatisfactory tanning efficiency (see below).

#### 3.3.2. AFRICA-Sudan: *Anogessus leiocarpus* Extract

Leaves and barks of *Anogessus leiocarpus,* grown in Sudan, could be an additional valuable resource to produce VTs. In fact, in a recent work, Haroun and coworkers [45] characterized the extracts of *Anogessus leiocarpus* leaves and barks and their efficiency as tanning agents. *Anogessus leiocarpus* leaves and barks were ground and extracted with water at different temperatures (between 35 °C and 85 °C) (Table 1). Both extracts obtained at 50 °C show a T/N ratio ≥ 1, although extracts of leaves gave better yield (%), T(%), TPC(%) and TFC(%) compared to bark extracts, suggesting that *Anogessus leiocarpus* could be suitable for vegetable tanning. No chemical characterization of the extract was performed; however, works in the literature [71,72] indicate that *Anogessus leiocarpus* contains both hydrolysable and condensed tannins. In the present study, however, it was compared to *A. mearnsii*, which is known to contain primarily condensed tannins.

#### 3.3.3. AFRICA-Ethiopia: *Cassia Singueana*, *Solanum incanum*, *Rumex abyssinicus*, *Eichhornia crassipes* and *Osyris lanceolata* Extracts

A considerable variety of new feedstocks for leather making have been reported from plants growing in Ethiopia, such as *Cassia singueana*, *Solanum incanum*, *Rumex abyssinicus*, *Eichhornia crassipes*, and *Hagenia abyssinica*, as described below. In Ethiopia, the leather industry employs many people, but cannot easily afford commercial VTs due to their high cost. The study of alternative sources of VTs, which are locally accessible, is therefore an important alternative to implementing VT leather production in this region.

Teklemedhin and coworkers [37] selected *Cassia singueana* bark, from a small shrub that is widely found in Africa [73], for the extraction of VTs and tanning efficiency of sheep skins compared to mimosa. Grinded *Cassia singueana* bark from Northern Ethiopia was extracted with water in different conditions (temperature, time), and extracts characterized by FT-IR, allowing it to be determined that *Cassia singueana* bark contains condensed tannins. A detailed study was carried out in order to identify optimal extraction concentrations (g/L), temperature and time. The best water extractions were obtained with a water solution concentration of 80 g/L at 100 °C for 120 min (yield 37 wt%, Table 1) and pH is 5.16, which is in the optimal range for tanning tests.

Another interesting cultivar from Ethiopia is Sodom apple, which is the common name for *Solanum incanum*, a wild plant that grows well in both hot and moderate climates. Seda Badessa and coworkers were the first to report on its potential use as a tanning agent [51]. In this study, tannin extraction was tested in different solvents (water, methanol, ethanol, and petroleum ether) and water was identified as the most effective and environmentally sustainable solvent, giving a yield of extraction of around 17 wt%. The extract was characterized using FT-IR, and phytochemical analysis showed the presence of condensed tannins, with a T/N ratio > 1 and pH between 4 and 6, confirming its suitability for leather tanning.

Mohammed and coworkers [52] explored the use of the herbal plant *Rumex abyssinicus* (mekmeko) for tanning applications. Mekmeko belongs to the *Polygonaceae* family, and is known to contain flavonoids, anthraquinones, and triterpenoids. The plant is widely distributed across Africa (especially in Ethiopia) and in Madagascar. In the work by Mohammed and coworkers, aqueous extraction of *Rumex abyssinicus* at 80 °C for 2 h was performed, yielding an extract with a T/N ratio > 1, indicating its potential for tanning. FT-IR and UV-Vis analysis confirmed the similarity to mimosa tannins with a polyphenolic structure.

Ahmed and coworkers verified the possibility of extracting VTs from stems and leaves of *Eichhornia crassipes* (water hyacinth), one of the most frequently infesting aquatic plants in the world, which is widely present in Ethiopia [53]. Extracts were obtained using three different solvents (water, acetone and ethanol), stirring leaf and stem powder at room temperature for 1 h. Phytochemical and FT-IR analysis evidenced a high concentration of flavonoids (condensed tannin) in *Eichhornia crassipes* extracts, and the tannin content of leaves (4.1%) was higher than that from stems (2.7%). Other important parameters such as T/N were not evaluated, but extracts were further tested as tanning agents as described below.

*Hagenia abyssinica,* widely available in Ethiopia, was studied by Unango and coworkers [54]. Extraction time, temperature, and solution concentration were studied, and the best yield and T(%) were obtained in water at 80 °C for 2 h after 48 h of soaking (23% and 15%, respectively). FT-IR spectroscopy verified that the extract consists of condensed tannins. A T/N ratio above 2 was achieved, due to the high content of condensed tannins.

Teshome and coworkers [55] tested *Osyris lanceolata* bark as a potential new source for VT for a chrome-free tanning process. *Osyris lanceolata* is described as a hardy evergreen shrub or small tree found in Ethiopia, the bark of which is reformed by the plant within a few months after peeling, making it a renewable resource. *Osyris lanceolata* bark was dried, ground and extracted in water at different temperatures and times. The best extraction conditions (65 °C for 120 min) allowed for obtaining more than 18% tannin yield with a T/N ratio of approximately 1. FT-IR and phytochemical analysis indicated the presence of condensed tannins.

#### 3.3.4. ASIA-Bangladesh: *Cassia fistula*, *Pontederia crassipes*, *Xylocarpus granatum* and *Azadirachta indica* Extracts

As in the case of Ethiopia, Bangladesh is also a geographical area in which various different cultivars have been tested for leather making. This of course is a consequence of the economic relevance of the leather industry in these geographical areas, and it would therefore highly benefit from the availability of innovative VT extracts derived from native plants, since VTs used in Bangladesh are mostly imported from faraway countries [74].

Oaishi and coworkers [56] tested the extraction of VTs from *Cassia fistula* bark with different solvents such as water, methanol, and ethanol. *Cassia fistula*, of the Fabaceae family, is grown almost everywhere in Bangladesh and is also present in South Africa, Mexico, East Africa, Thailand, India, China, the Philippines, Malaysia, and Indonesia [75], and could be a viable alternative for VTs for the leather tanning industry. Ground *Cassia fistula* bark was extracted with different solvents at room temperature for 2 h, and the solution was dried to recover 21 wt% from ethanol, 16 wt% from methanol and only 4 wt% from water. Further characterisations were carried out on the fraction extracted with ethanol. UV-Vis and FTIR analysis confirmed that the VT extracted from *Cassia fistula* bark is catechin-based.

Additionally, Mustafa and coworkers [57] studied the characteristics of *Pontederia crassipes* extracts obtained with various solvents at room temperature for 6 h (water, acetone, ethanol and methanol), showing that methanol gave the best results, with yields up to 26 wt% and more than 10 wt% of tannins. Further characterisations revealed that the methanol extract contained both condensed and hydrolysable tannins in different amounts (22–25% and 12–14%, respectively).

Another interesting feedstock is the bark of *Xylocarpus granatum*, which contains tannins and a reddish-brown dye. Studies have shown that the bark of various mangrove species can contain between 16 wt% and 48 wt% tannins [5]. Das and coworkers [58] reported FT-IR, UV-Vis and HPLC characterisations and tanning efficiency of tannins extracted from *Xylocarpus granatum* bark and assessed its potential for commercial use as a novel source of condensed tannins. Ethanol and methanol extractions yielded the best results (over 30 wt%), but the pH (4) of the methanol extract was more suitable for tanning. The total tannic content (mg of tannic acid equivalent/g of dry extract) was similar to those contained in commercial VTs such as mimosa and quebracho (see Table 1).

Several studies have highlighted that *Azadirachta indica* (neem tree) leaves have minimal non-tannin components and could be a valuable source of natural tanning agents, as reported by Shakil and coworkers [59]. Leaf powder was obtained by grinding leaves, while leaf extract was made by shaking leaves with different solvents (acetone, water, ethanol and methanol) for 2 h at 70 °C. The best extraction yield was reached using ethanol (15.5 wt%), and tannin content was identified qualitatively and quantitatively. Leaf powder was also characterized, but a lower tannin content was identified (10 wt% versus 12 wt% from leaf extracts). Both *Azadirachta indica* leaf powder and leaf extract contain condensed tannins, as confirmed by FT-IR analysis.

#### 3.3.5. ASIA-Indonesia: *A. mangium* and *A. auriculiformis* Extracts

In Indonesia, there are several companies using *A. mangium* and *A. auriculiformis* as raw material for pulp and paper production, but the bark contains tannins that could be used as tanning agents. Extractions at 100 °C in water showed that the yield of *A. auriculiformis* bark was higher than that of *A. mangium* bark extract, and its T(%) reached more than 11% [60].

#### 3.3.6. ASIA-Pakistan: *Eucalyptus globulus* Extracts

Khan and coworkers [61] selected and tested *Acacia Nilotica* L. and *Eucalyptus globulus*, which are abundantly available in Pakistan. *A. Nilotica* L. has already been studied several times, and was therefore considered as a reference [31,38,61,76,77,78]. Various extraction techniques and solvents were tested, and it was concluded that acetone–water solvent mixtures in a 70/30 ratio and ultrasonic extraction were the best conditions to obtain maximum tannin content (196 mg/g for *A. nilotica* L. and 125 mg/g for *Eucalyptus globulus*). FT-IR analysis confirmed the presence of condensed tannins in both samples.

### 3.4. Hydrolysable Tannins

#### ASIA-China: *Coriaria nepalensins* Extract

Recently, Guo and coworkers [62] investigated the applicability of tannins obtained from the *Coriaria nepalensins* bark (CNB, hydrolysable tannin), as well as the best extraction conditions. CNB extraction tests were carried out in the presence of variable concentrations of NaOH at different temperatures and times, and the yield of purified CNB tannin extracts was measured. The optimal extraction condition was obtained at 82 °C for 63 min using 0.22% of NaOH with an extraction yield of 16 wt%. Extracts were further characterized (^1^H and ^13^C NMR spectroscopy and GPC), establishing the hydrolysable nature of the tannin. In addition, CNB extract was found to have a very high tannin content (more than 42%), confirming its potential for leather tanning.

## 4. Characteristics and Applications of Leather Produced with Different Vegetable Tanning Systems

The quality of the leathers tanned with the different VTs reported above has been verified according to conventional tests to determine different physical–mechanical properties such as tensile strength (TS), elongation at break (EB%), tear strength (Ts) and softness, together with hydrothermal stability (HS) (Table 2, Table 3 and Table 4). HS is often employed to verify tanning efficiency, as this parameter is particularly important in determining the processability of tanned leather, which should be at least above 75 °C to allow for mechanical processing such as trimming and splitting operations [5]. Raw hides and skins naturally exhibit an HS of approximately 60–62 °C, which is generally increased to temperatures ≥ 80 °C after VTs tanning. This enhancement is attributed to the cross-linking between collagen and polyphenolic compounds present in the extract, by means of hydrogen bonding and secondary interactions with free amine and hydroxyl groups present in the collagen matrix. HS is an indirect indicator of the strength of covalent bonds or hydrogen and Van der Waals interactions formed within the collagen matrix. Stronger bonds between the collagen protein and the tanning agent generally result in higher HS, whereas weaker bonds lead to lower HS [30,79,80]. Consequently, HS is an essential measure of both the extent of collagen bonding and the overall quality of tanned leather [5].

This parameter can be expressed by two different temperatures: the shrinkage temperature (T_s_) and denaturation temperature (T_d_).

T_s_ is typically measured by immersing a leather strip in water and slowly heating it, observing the point at which it suddenly contracts. IUP16 and ISO 3380:2015 are standard methods for determining this temperature [30,81].

Differential scanning calorimetry (DSC), instead, is often used to measure T_d_, where it refers to the temperature at which the collagen protein in leather undergoes a structural change from its organized triple-helix form to a more disordered random coil structure [5].

VTs may be used in different phases of the process as pretanning, tanning or retanning agents, generally in combination with other tanning agents (aldehydes, chrome salts, among others). The different protocols reported in the 15 articles reviewed in this paper have been described below in different sections depending on the use the VT is reported for and not by geographical origin. Additional information on the processing conditions employed in the papers reviewed in reported in the supporting info section.

### 4.1. Vegetable Extract Employed for Tanning Tests

China and coworkers [50] performed preliminary crosslinking tests with hide powder to compare the efficiency of commercially available *A. mearnsii* with that of the new extracts from *A. xanthopea*, *E. divinorum* and *E. racemosa*.

First, hide powder was treated with 5 wt% of the different plant extracts at increasing pH (between 3 and 8) and tanning efficiency determined by DSC. These data show a significant influence of the pH on the efficiency of the different extracts, and in particular *A. mearnsii* generated a maximum increase in T_d_ (dT_max_) of about 25–26 °C at pH 4, similarly to *E. divinorum* (dT_max_ 24–25 °C, pH 4), while *A. xanthopea* gave slightly higher dT (dT_max_ 30 °C, pH 4). On the other hand, *E. racemosa* gave very little dT, not higher than 5 °C, probably due to the low content of tannins present in this extract, and was therefore not further tested. China and coworkers suggest that the behaviour of the tested extracts at variable pH may indicate that hydroxyl groups (at acidic pH) and ketonic carbonyl groups (at alkali pH) are able to interact with protonated and unprotonated amino groups via hydrogen and covalent bonds, respectively, which is typical for condensed tannins [70].

Leather tanned with *A. xanthophloea* gave similar TS results to that tanned with *A. mearnsii*, but better EB%. Its softness is slightly lower than *A. mearnsii*, though not by much. In contrast, leather tanned with *E. divinorum* extracts performs worse across all mechanical measures. This is likely due to *E. divinorum*’s lower content of tanning agents, total phenolics, and flavonoids, which impairs its ability to properly crosslink collagen molecules.

#### 4.1.1. *Anogessus leiocarpus*

Haroun and coworkers [45] used extracts obtained from leaves and bark of *Anogessus leiocarpus* as potential tanning agents and compared the results with *A. mearnsii* bark-tanned leather. With the conventional tannin method, limed pelts were tanned with 22 wt% of each extract. Mechanical properties of tanned leather with *Anogessus leiocapurs* leaves were comparable to those obtained using *A. mearnsii*; in particular, TS and EB% were very similar (10 N/mm^2^ and 40%, respectively; see Table 2).

On the other hand, leather tanned with *Anogeissus leiocarpus* leaf extracts exhibited slightly lower softness values compared to *A. mearnsii*, although the difference was not substantial. In contrast, leather tanned with *Anogeissus leiocarpus* bark extracts showed consistently lower values across all parameters when compared to *A. mearnsii* (see Table 2). These results support the current study’s findings, which confirm the limited ability of *Anogeissus leiocarpus* bark extract to crosslink collagen molecules effectively, likely due to its lower tanning capacity and reduced total phenolic and flavonoid content, ultimately impacting the mechanical properties of the leather.

Interestingly, the T_s_ of leather tanned with *Anogessus leiocapurs* (90 °C) leaves was comparable to that achieved with *A. xanthophloea* [50] and higher than *A. mearnsii* bark (85 °C).

#### 4.1.2. *Cassia singueana*

Teklemedhin and coworkers [37] employed 25 wt% of *Cassia fistula* bark extracts for sheep skin tanning (see Table S1), and the results were compared with those obtained in similar tanning conditions employing commercially available mimosa extracts. T_s_ and physical mechanical properties (TS, EB% and Ts) of both extracts were very similar, demonstrating that *Cassia singueana* is a good environmentally sustainable alternative to mimosa extracts. In particular, all mechanical properties tested are slightly higher than mimosa extract (T_s_ > 80 °C, see Table 2). In addition, EB% and Ts fulfilled the minimum values set by IUP6 and IUP8 standard (>40% and >20 N/mm) [81,82].

#### 4.1.3. *Solanum incanum*

Goat skin was used by Seda Badessa and coworkers [51] to evaluate the tanning potential of *Solanum incanum* and the results were compared with commercially available mimosa extract (see Table S2). Briefly, 25 wt% of each VT was used in the tanning process and leather obtained characterized. Scanning Electron Microscope (SEM) analysis was used to examine the fibre compactness and grain surface of VTs tanned leathers and revealed a stronger association between *Solanum incanum* and goat skin compared to mimosa. Mechanical properties were also evaluated to establish the quality of leather obtained. TS, EB% and Ts were higher with respect to mimosa-tanned leather and in line with the requirements of IUP6 and IUP8 [81,82]. T_s_ was slightly lower with respect to mimosa-tanned leather (75 °C and 78 °C, respectively).

#### 4.1.4. *Acacia mangium* and *Acacia auriculiformis*

In a study by Mutiar and Kasim [60], goat skins were tanned using bark extracts from *A. mangium* and *A. auriculiformis*. While the precise amounts of extract used were not specified, the tanned leather’s physical properties were assessed against those achieved with mimosa or quebracho. Both novel tanning agents yielded leather with a higher EB%, and *A. auriculiformis* notably exhibited favourable TS compared to the commercial VTs. A key limitation was the lower wrinkle temperature observed for both new extracts; specifically, *A. mangium* did not meet the United Nations Industrial Development Organization (UNIDO) recommended minimum of 75 °C. Despite this, the authors reported that the results were consistent with the Indonesian National Standard.

**Table 2:** Physical–mechanical properties of leather tanned with VTs.

VT Name	Hide	Wt%	HS (°C)	Tensile Strength (N/mm^2^)	Elongation at Break (%)	Tear Strength (N/mm)	Softness (mm)	Methods	Ref.
			>75	>22.55	30–45	>29.4		UNIDO 1996 ^a^ [83]	
**VTs employed for tanning tests**									
*A. xanthoplea*	hide powder	22	92	5.69 ± 0.3	36.80 ± 1.89	5.69 ± 0.3	1.9 ± 0.4	EN ISO 17235:2015 ^b^ [84]	[50]
*E. divinorum*			88	4.71 ± 1.0	26.30 ± 1.41	4.71 ± 1.0	1.7 ± 0.4		
***A. mearnsii***			86	6.06 ± 0.6	33.44 ± 1.98	6.06 ± 0.6	2.4 ± 0.3		
*Anogessus leiocarpus (leaves)*	pelt	22	90	10.5 ± 0.35	40.6 ± 0.4		2.6 ± 0.5		[45]
*Anogessus leiocarpus (bark)*			86	7.8 ± 0.25	32.0 ± 0.6		1.5 ± 0.8		
***A. mearnsii***			89	10 ± 0.35	40 ± 0.35		1.0 ± 0.05		
*Cassia singueana*	sheep	25	83	15.6	45.3	24.2		IUP6 ^c^, IUP8 ^d^, IUP16 ^e^ [81,82,83]	[37]
***Mimosa***			80	14.8	38.7	22.5			
*Solanum incanum*	goat	25	75	14.2	43.7	23.4		IUP6 ^c^, IUP8 ^d^, IUP16 ^e^ [81,82,83]	[51]
***Mimosa***			78	12.5	30.2	21.5			
*A. mangium*	goat		74	21.03	38.26				[60]
*A. auriculiformis*			80	24.30	39.60				
***Mimosa***			82	23.96	26.02				
***Quebracho***			82	17.28	28.92				

(a) UNIDO 1996: UNIDO. Acceptable quality standards in the leather and footwear industry, 1996; (b) EN ISO 17235:2015: Leather—Physical and mechanical tests—Determination of softness; (c) IUP6: International Union for Physical Testing: Measurement of tensile strength and percentage elongation; (d) IUP8: International Union for Physical Testing: Measurement of tear load; (e) IUP16: International Union for Physical Testing: Measurement of shrinkage temperature up to 100 °C.

### 4.2. Vegetable Extract Employed for Tanning and Retanning Tests

#### 4.2.1. *Hagenia abyssinica*

Unango and coworkers tested the efficiency of *Hagenia abyssinica* extracts from African trees with commercially available mimosa extracts for the tanning of pickled sheep skins [54]. Skins were tanned with 8 wt% in three portions (24 wt% in total) of the extracts by weight of hide and retanned, dyed and finished according to the protocol reported in Table S3. Crust upper leathers, tanned and retanned with *Hagenia abyssinica* extract, were characterized according to IUP methods (IUP2, IUP6, IUP8 and IUP16) [81,82,85,86], and TS, EB% and Ts were compared to mimosa tanned and retanned leather. Neither crust leather sample met the UNIDO requirements for TS and Ts and, in particular, the quality of *Hagenia abyssinica* crust leather seems relatively unsatisfactory, showing that *Hagenia abyssinica* does not appear to be a commercially sustainable alternative for leather manufacture. In particular, Unango and coworkers affirmed that the leather samples absorbed too many fats from the pretanning step, causing a reduction in tannin penetration.

#### 4.2.2. *Pontederia crassipes*

*Pontederia crassipes* and mimosa extracts were tested by Mustafa and coworkers for the tanning and retanning of goat skin, and the data compared [57]. For further detail on the tanning and retanning protocol used, see Table S4. The *Pontederia crassipes* tanned and retanned leather showed enhanced hydrothermal stability with a T_s_ of 83.3 °C, comparable to that of conventional mimosa retanned leather (82.2 °C). This indicates effective collagen–polyphenol bonding and confirmed that *Pontederia crassipes* meets the thermal stability standards for practical leather applications. The physical–mechanical evaluation of the *Pontederia crassipes* crust leather treated showed a significant enhancement in TS (28.08 N/mm^2^), surpassing both the UNIDO standard (22.55 N/mm^2^) and conventionally mimosa tanned leather (26.15 N/mm^2^, see Table 2). This significant improvement may be attributed to the unique chemical composition of *Pontederia crassipes* tannins, containing both condensed (22–24%) and hydrolysable tannins (12–14%), improving cross-linking with collagen fibres and therefore enhancing TS. Moreover, an EB% of 63.33% was measured, which is superior to most commercially available VTs, indicating that *Pontederia crassipes* tanned leather has high flexibility and load-bearing capacity. The observed improvements in these physical–mechanical properties suggest the potential of *Pontederia crassipes* extracts as a promising alternative for superior leather processing.

#### 4.2.3. *Xylocarpus granatum*

Das and coworkers compared the performances of *Xylocarpus granatum* bark to conventional condensed tannins such as mimosa and quebracho [58]. Goat skins were tanned and retanned with overall 21 wt% of *Xylocarpus granatum* bark extracts to give a retanned leather with T_s_ higher than conventional VTs, suggesting that *Xylocarpus granatum* bark extracts could substitute conventional mimosa and quebracho tannins in terms of hydrothermal stability. Physical–mechanical properties (TS, EB% and tear strength) were above the UNIDO standard for upper leather production and comparable to conventional VT tanned leather (Table 3). This suggests that *Xylocarpus granatum* tannin could be a good alternative to conventional commercially available tanning agents in geographical areas where it is abundant, reducing transportation-based environmental impacts and costs (Bangladesh and tropical areas of East Africa, Polynesia, Thailand, Indonesia, Myanmar, Malaysia, India, China, and Australia).

#### 4.2.4. *Azadirachta indica*

*Azadirachta indica* leaf powder and related extracts obtained as reported above (Section 3.3.4) were tested with goat skins as tanning and retanning agents (Table S6) and their efficiency compared to mimosa-tanned and retanned crust leather [59]. The T_s_ of leather tanned with overall (tanning and retanning) 28 wt% of *Azadirachta indica* leaf powder, extract, and mimosa was found to be 81 °C, 86 °C, and 84 °C, respectively. Although crust leather obtained using *Azadirachta indica* leaf powder was harder (tear strength 146 N/mm) and less flexible (EB% 38.7%, see Table 3), the overall physical–mechanical characteristics of crust leather obtained with *Azadirachta indica* leaf powder and extract were generally comparable to those of mimosa (Table 3). The extract of *Azadirachta indica*, instead, exhibited lower performance compared to leaf powder in terms of TS and Ts but met the minimum standard values. The authors concluded that differences between leaf powder and extracts allow two potential tanning agents to be used for different types of leather goods.

#### 4.2.5. *Eucalyptus globulus*

Using pickled goat hides (see Table S7), Khan and coworkers investigated the potential of tannin extracts from the bark of *E. globulus* as environmentally friendly tanning (350 mL) and retanning (350 mL) agents and compared their efficiency with mimosa and *A. Nilotica*, commonly employed in Africa [31,38,61,76,77,78]. Crust leather T_s_, TS and EB% were measured according to IUP6 standard methods [81], and the results evidence that *E. globulus* crust leather has lower T_s_ with respect to *A. Nilotica* but reached the minimum standard request value (75 °C). Other physical–mechanical properties were comparable to *A. Nilotica* and mimosa leathers, demonstrating that *E. globulus* may be employed as a substitute for commercially available tannins.

#### 4.2.6. *Coriaria nepalensis*

Guo and coworkers studied the tanning and retanning efficiency of *Coriaria nepalensis* (20 wt% by weight of hides) on delimed sheep skin with a conventional tanning protocol (see Table S8), achieving T_s_ slightly superior to that of commercially available Valonia (76.4 and 72.1 °C, respectively) [62]. Additionally, TS (6.1 N/mm^2^), EB% (75.8%) and Ts (38.2 N/mm) were also moderately higher than those achieved with Valonia (see Table 2). Finally, SEM analysis confirmed that Coriaria Nepalensis provides a delicately grained surface, demonstrating that Valonia can be substituted by Coriaria Nepalensis in those geographical areas where it is abundant (China and Indian sub-continent) [87].

**Table 3:** Physical–mechanical properties of leather tanned and retanned with VTs.

VT Name	Hide	Wt%	HS (°C)	Tensile Strength (N/mm^2^)	Elongation at Break (%)	Tear Strength (N/mm)	Softness (mm)	Methods	Ref.
			>75	>22.55	30–45	>29.4		UNIDO 1996 ^a^ [83]	
**VTs employed for tanning and retanning tests**									
*Hagenia abyssinica*	sheep	24 (tanning) 4 (retanning)	73.3	10.25 ± 0.03	45.71 ± 0.02	26.90 ± 0.04		IUP2 ^b^, IUP6 ^c^, IUP8 ^d^, IUP16 ^e^ [81,82,85,86]	[54]
***Mimosa***		15 (tanning) 4 (retanning)	75.1	19.8 ± 0.03	36.72 ± 0.03	27.82 ± 0.02			
*Pontederia crassipes*	goat	30 (tanning) 15 (retanning)	83.3	28.08 ± 1.35	63.33 ± 4.16	40.5 ± 0.8		IUP6 ^c^, IUP8 ^d^ [81]	[57]
***Mimosa***		30 (tanning) 15 (retanning)	82.2	26.15 ± 1.21	52.6 ± 3.41	33.7 ± 0.5			
*Xylocarpus granatum*	goat	3 (tanning) 18 (retanning)	86.34	29.17 ± 1.39	42.545 ± 2.05	38.2 ± 4.5		IUP6 ^c^, IUP8 ^d^ [81,82]	[58]
***Mimosa/Quebracho***		3 (tanning) 24 (retanning)	81.34	25.36 ± 1.61	38.675 ± 3.57	36.5 ± 3.3			
*Azadirachta indica* *(powder)*	goat	8 (tanning) 20 (retanning)	81	29.2	38.72	146		IUP6 ^c^, IUP8 ^d^, IUP16 ^e^ [81,82,85]	[59]
*Azadirachta indica* *(extract)*		8 (tanning) 20 (retanning)	86	27.71	40.71	144			
***Mimosa***		8 (tanning) 20 (retanning)	84	29.42	37.29	141			
*E. globulus*	goat	350 mL (tanning) 350 mL (retanning)	75	35	74			IUP6 ^c^ [81]	[61]
***A.nilotica***		350 mL (tanning) 350 mL (retanning)	80	38	85				
***Mimosa***		350 mL (tanning) 350 mL (retanning)	76	42	58				
*Coriaria nepalensis*	sheep	2 (pretanning) 18 (tanning)	76.4	6.1	75.8	38.2			[62]
***Valonia***		2 (pretanning) 18 (tanning)	72.1	5	68	38			

(a) UNIDO 1996: UNIDO. Acceptable quality standards in the leather and footwear industry, 1996; (b) IUP2: International Union for Physical Testing: Leather—Chemical, physical and mechanical and fastness tests—Sampling location. (c) IUP6: International Union for Physical Testing: Measurement of tensile strength and percentage elongation; (d) IUP8: International Union for Physical Testing: Measurement of tear load; (e) IUP16: International Union for Physical Testing: Measurement of shrinkage temperature up to 100 °C.

### 4.3. Vegetable Extract Employed for Leather Tanning Followed by Synthetic Retanning

#### 4.3.1. *Eichhornia crassipes*

Ahmed and coworkers reported the use of water hyacinth leaves in tanning tests, while stem extracts were not tested due to their lower tannin content (see above) [53]. Different tests were performed using 10 wt% and 20 wt% of *Eichhornia crassipes* leaves alone or in combination with quebracho (5 wt% water hyacinth leaves and 5 wt% quebracho) and data compared with leather tanned with 10 wt% quebracho (Table 4). All tanning tests were followed by conventional syntan retanning (see Table S9). Data reported show that hides tanned with 10 wt% quebracho had the highest T_s_ (78 °C), whereas those tanned with 10 wt% and 20 wt% *Eichhornia crassipes* reached very low T_s_ (55 °C and 52 °C, respectively), and also combination tanning had unsatisfying T_s_ (58 °C), much lower than the minimum acceptable shrinkage temperature (75 °C) for leather production. Also, TS and EB% were unfortunately below the minimum requirements for upper leather production, even if 5 wt% quebracho was used in combination with an equivalent amount of *Eichhornia crassipes* followed by synthetic retanning (see Table 4 and Table S9). As far as the EB% values are concerned, it should be mentioned that hide tanned with 10 wt% and 20 wt% *Eichhornia crassipes* leaves, as well as in combination with quebracho, reached values ≥ 40% for percentage elongation, and therefore met the requirements for upper leather production, in agreement with the standard requirements (UNIDO). Overall, in contrast to other condensed tannin extracts reported, *Eichhornia crassipes* is not a sustainable alternative for leather manufacturing.

#### 4.3.2. *Cassia fistula*

Oaishi and coworkers [56] tested *Cassia fistula* bark extracts for the tanning (4 wt% by wt of skin) and retanning (18 wt% of *Cassia fistula* bark extract and 18%wt of a syntan) of goat skins (see Table S10) and measured T_s_ and physical–mechanical characteristics (see Table 2). A T_s_ of 86 °C was obtained according to IUP 16 standard method [81], and physical–mechanical characteristics of crust leather obtained were measured. All physical–mechanical properties complied with the requirements of the UNIDO standard; in particular, TS (>27 N/mm^2^) EB% (>38%) and Ts (>43 N/mm) were above required minimum standard values, making this tannin source even more attractive from an economic and practical point of view. It should nevertheless be considered that the *Cassia fistula* bark extracts tested were obtained by ethanol extraction, which limits the environmental sustainability and industrial viability of this specific feedstock (yield of water extracts was only 4 wt%).

#### 4.3.3. *Rumex abyssinicus*

Mohammed and coworkers [52] tested *Rumex abyssinicus* tanning efficiency at different concentrations (5–30 wt%) on pickled goat skins combined with 2 wt% of a phenolic syntan (Table S11). Results were compared to a control using 15 wt% mimosa extract (Table 4). The study also included a retanning test with synthetic tanning agents and measured the extracts’ fixation. The T_s_ of tanned leather increased with increasing concentration of the *Rumex abyssinicus* extract up to 20 wt%, while no significant increase in T_s_ (77.0 ± 2.0) and extract fixation (72 ± 2.0) was observed if higher concentrations were employed. Also, in this case, the pH of the extract plays a significant role in the absorption of tannins and highest T_s_ and float exhaustion are achieved at pH 5. TS, EB% and Ts of crust leather was comparable to the control sample, with improved softness, confirming that *Rumec abyssinicus* extract is a greener alternative for leather manufacturing within the principles of proximity economy (Table 4).

#### 4.3.4. *Osyris lanceolata*

Sheep pickled pelt was used to test the tanning efficiency of *Osyris lanceolata* bark extracts [55]. Pelts were pre tanned with 4 wt% of a synthetic tannin, followed by treatment with 30 wt% *Osyris lanceolata* and retanning with a syntan (3%wt) as reported in Table S12. Crust leather had good T_s_ (84.5 °C), with TS and EB% in agreement with the minimum standard values.

**Table 4:** Physical–mechanical properties of leather tanned with VTs and retanned with synthetic retanning agents.

VT Name	Hide	Wt%	HS (°C)	Tensile Strength (N/mm^2^)	Elongation at Break (%)	Tear Strength (N/mm)	Softness (mm)	Methods	Ref.
			>75	>22.55	30–45	>29.4		UNIDO 1996 ^a^ [83]	
**VTs employed for tanning and synthetic retanning tests**									
*Eichhornia crassipes*	-	10 (tanning) 8 Retanal MD 80/LSF-100 (retanning)	55	6	47	37		UNIDO 1996 ^a^ [83]	[53]
		20 (tanning) 8 Retanal MD 80/LSF-100 (retanning)	52	7.2	42	45			
		5 + 5 (Quebracho) (tanning) 8 Retanal MD 80/LSF-100 (retanning)	58	8.9	60.5	106.2			
***Quebracho***		10 (tanning) 8 Retanal MD 80/LSF-100 (retanning)	76	22	62	115			
*Cassia fistula*	goat	4 (tanning) 18 + 18 (syntan) (retanning)	85.77	27.61 ± 0.54	38.02 ± 1.79	43.1 ± 0.45		UNIDO 1996 ^a^, IUP16 ^b^ [81,84]	[56]
*Rumex abyssinicus*	goat	5–30 (tanning) 6 (syntan) (retanning)	61–78	20.82 ± 0.29	48 ± 0.7	39.2 ± 0.9		IUP6 ^c^, IUP8 ^d^ [81,82]	[52]
***Mimosa***		15 (tanning) 6(syntan) (retanning)	80	20.59 ± 0.20	48.5 ± 1.4	39.2 ± 0.9			
*Osyris lanceolata*	sheep	30 (tanning) 3 (Basyntan D) (retanning)	84.5	12.36	42.6	15–18 ^e^			[55]

(a) UNIDO 1996: UNIDO. Acceptable quality standards in the leather and footwear industry, 1996; (b) IUP16: International Union for Physical Testing: Measurement of shrinkage temperature up to 100 °C; (c) IUP6: International Union for Physical Testing: Measurement of tensile strength and percentage elongation; (d) IUP8: International Union for Physical Testing: Measurement of tear load; (e) force measured in N.

## 5. Environmental Impact

Only two of the papers reported above evaluated the environmental impact of the new extracts in comparison with those commercially available. In particular, Teklemedhin and coworkers [37] compared the chemical oxygen and biological oxygen demand (COD and BOD), and total dissolved solids (TDS) of wastewater collected after leather tanning with *Cassia singueana* or commercially available mimosa. From these data, it emerges that COD, BOD and TDS in wastewater collected after tanning with both extracts were equivalent (Table 5). Unango and coworkers [54] found similar results for the tanning liquors from *H. abyssinica* and mimosa tanning. Interestingly, data reported in these studies are completely different, especially for conventional tannins, yet overall they evidence that commercially available tannins and the new extracts are equivalent in terms of environmental impact.

## 6. Conclusions

The scope of this review is to provide an overview on new extracts coming from different cultivars and geographical areas and their efficiency for leather processing. Within the boundaries set by the Green Deal and the UN Agenda 2030, the industry is seeking alternative sustainable best practices to reach carbon neutrality by 2050. In this panorama, the possibility of finding alternative natural feedstocks, available locally, is an interesting solution to reduce greenhouse gas production and improve the carbon footprint of the leather industry. At present, most vegetable tannins employed for leather processing come from limited geographical areas, and their transportation highly impacts the overall production chain. This review provides a comprehensive overview of the potential of alternative vegetable tannins (VTs) derived from non-conventional plant sources across different geographical regions for application in the leather industry. The systematic analysis of the recent literature demonstrates that several plant species—particularly those endemic to Africa and Asia—yield tannin-rich extracts with physicochemical and mechanical properties comparable to or even exceeding those of conventional commercial tanning agents such as mimosa and quebracho.

Key parameters such as the tannin-to-non-tannin ratio (T/N), tannin content (T%), total phenolic content (TPC), total flavonoid content (TFC), extraction yield and pH were identified as crucial indicators of tanning efficiency and extract quality. Extracts with a T/N > 1, such as those obtained from *Acacia xanthophloea*, *Cassia singueana*, *Solanum incanum*, *Pontederia crassipes*, and *Xylocarpus granatum*, demonstrated strong collagen crosslinking capacity (high HS) and favourable mechanical properties, including tensile strength, elongation at break, and tear strength. Moreover, preliminary data on environmental parameters (e.g., COD, BOD, and TDS) suggest that these novel extracts have an environmental impact comparable to traditional VTs.

However, the effectiveness of these alternative sources is highly dependent on factors such as plant species, plant part used, geographical origin, extraction conditions, and seasonal variability. In some cases—such as *Eichhornia crassipes* and *Hagenia abyssinica*—the mechanical and thermal properties of the resulting leather did not meet industry standards, highlighting the need for further optimization and evaluation.

Overall, the valorisation of locally available, renewable plant resources for VT production offers a promising strategy to enhance proximity-based economies, reduce transportation-related emissions, and support the development of sustainable leather manufacturing practices. Future research should focus on refining extraction protocols, improving the consistency of tannin-rich extracts, and validating their scalability in industrial tanning processes. These efforts are essential for fostering the transition toward more sustainable, chrome-free, and regionally integrated leather production systems.

## Figures and Tables

**Table 1:** Botanical and common name, geographical origin, T/N, T(%), TPC(%), TFC(%), yield(%), ash(%), and pH of different tannins.

Botanical Name	Common Name	Geographical Origin	T/N ^(a)^	T (%)	TPC (%) ^a^	TFC (%) ^a^	Yield (%) ^a^	Ash (%)	pH	Ref.
**CONDENSED TANNINS**										
***Africa***										
*A. xanthophloea*	Fever tree	Northern Tanzania	6.6	33	13	10	40 ^b^			[50]
*E. divinorum*	Diamond leaf	Northern Tanzania	0.9	11	3	2	16 ^b^			
*E. racemosa*	Sea guarrie	Northern Tanzania	0.3	7	2	2	47 ^b^			
***A. mearnsii***			7.7	47	18	12	55 ^b^			
*Anogessus leiocarpus*	African birch (leaves)	Sudan	1.3	16	22.4	30.7	38 ^b^			[45]
*Anogessus leiocarpus*	African birch (barks)	Sudan	1.1	10	9.6	12.7	17 ^b^			
*Cassia singueana*	Winter cassia	Northern Ethiopia		37			37 ^c^	3.3	5.2	[37]
*Solanum incanum*	Sodom apple	Southern Ethiopia	1.3	12			17 ^d^		5.2	[51]
*Rumex abyssinicus*	Mekmeko	Ethiopia	1.1	18						[52]
*Eichhornia crassipes*	Water hyacinth (leaves)	Ethiopia		4				14.8	6.3	[53]
*Eichhornia crassipes*	Water hyacinth (stems)	Ethiopia		3				15.6	5.8	
*Hagenia abyssinica*	African redwood	Ethiopia	2.1	15			23 ^e^		4.7	[54]
*Osyris lanceolata*	African sandalwood	Ethiopia	0.9	18	20				4.7	[55]
***Asia***										
*Cassia fistula*	Golden shower tree	Bangladesh			159 ^f^				4.0	[56]
*Pontederia crassipes*	Water hyacinth	Bangladesh		>10			26 ^g^		4.2	[57]
*Xylocarpus granatum*	Dhundul	Bangladesh		121 ^h^			31 ^i^		4	[58]
	**Mimosa**			114 ^h^					4.8	
	**Quebracho**			130 ^h^					4.9	
*Azadirachta indica*	Neem tree (leaf powder)	Bangladesh	0.6	10					4.9	[59]
*Azadirachta indica*	Neem tree (leaf extract)	Bangladesh	1.8	12					4.8	
	**Mimosa**		2.38	64					4.9	
*A. mangium*	Silver wattle	Indonesia		11			24 ^j^			[60]
*A. auriculiformis*	Silver wattle			12			26 ^j^			
*Eucalyptus globulus*	Eucalyptus	Pakistan		125 ^k^	199 ^l^					[61]
***A. Nilotica* L**.				196 ^k^	319 ^l^					
**HYDROLYSABLE TANNINS**										
*Asia*										
*Coriaria nepalensis*	Masuri berry	China	0.8	43			16 ^m^	7.5		[62]
	**Valonia**									

(a) T/N: tannin/non tanning ratio; TPC (%): Total Phenol Content (%); TFC (%): Total Flavonoid Content (%);Yield (%): Total extraction yield (%). (b) Yield of VT extracted at 50 °C in 4 h; (c) Yield of VT extracted at 100 °C in 2 h. (d)Yield of VT extracted in 12 h. (e) Yield of VT extracted at 80 °C in 2 h after 48 h soaking. (f) mg of gallic acid equivalent/100 g of extracted sample. (g) Yield of VT extracted at 25 °C in 6 h in methanol. (h) mg of tannic acid equivalent/g of dry extract. (i) Yield of VT extracted at 25 °C in 11 h in methanol. (j) Yield of VT extracted at 100 °C in 30 min. (k) Hide Powder method mg/g (l) mg of gallic acid equivalent/g bark. (m) Yields of VT extracted at 82 °C in 63 min with 0.22% NaOH solution.

**Table 5:** COD, BOD and TDS of new and conventional VTs.

VT Name	BOD (mg/L)	COD (mg/L)	TDS (mg/L)	Refs.
*Cassia singueana*	1.17 ± 0.08 ^a^	3.35 ± 0.03 ^a^	18.21 ± 0.07 ^a^	[37,88,89]
**Mimosa**	1.24 ± 0.07 ^a^	3.47 ± 0.05 ^a^	22.63 ± 0.08 ^a^	
*H. abyssinica*	3.90 ± 0.05 ^b^	7.35 ± 0.85 ^b^	59.45 ± 1.15 ^b^	[54,90]
**Mimosa**	3.85 ± 0.05 ^b^	8.30 ± 0.20 ^b^	58.70 ± 1.80 ^b^	

(a) Apha, *Standard Methods for the Examination of Water and Wastewater*, Apha, American Public Health Association, Washington, D.C., USA, 1985; L. S. Clesceri, A. E. Greenberg, and R. R. Trussell, *“American Public Health.” American Water Works, Water Pollution Control, Standard Methods for the Examination of Water and Wastewater*, American Public Health Association, Washington, DC, USA, 17th edition, 1989. (b) AOAC. (1996). Association of Official Analytical Chemists (AOAC) manual. Rockville, MD 20850.

## Data Availability

The original contributions presented in this study are included in the article. Further material is available on request.

## Supplementary Materials

The following supporting information can be downloaded at https://www.mdpi.com/article/10.3390/ma18163759/s1, Table S1. Recipe for tanning of sheep pickle pelt using *Cassia singueana* and mimosa extract; Table S2. Recipe for tanning of goat skin using *Solanum incanum* and mimosa extract; Table S3. Recipe for tanning sheep skin using *Hagenia abyssinica* and mimosa extract; Table S4. Recipe for tanning of goat skin using *Pontederia crassipes* and mimosa extract; Table S5. Recipe for tanning goat skin using *Xylocarpus granatum* and mimosa extract; Table S6. Recipe for tanning of goat skin using *Azadirachta indica* and mimosa extract; Table S7. Recipe for tanning of goat skin using *Eucalyptus globulus* and *A. Nilotica* extract; Table S8. Recipe for tanning sheep skin using *Coriaria nepalensis* and valonia extract; Table S9. Recipe for tanning of skin using *Eichhornia crassipes* (10 %wt and 20%wt), combination of *Eichhornia crassipes* and quebracho (5 %wt + 5 %wt) and quebracho (10 %wt); Table S10. Recipe for tanning goat skin using *Cassia fistula* extract; Table S11. Recipe for tanning of goat skin using *Rumex abyssinicus* and mimosa extract; Table S12. Recipe for tanning sheep skin using *Osyris lanceolata* extract.

## Abbreviations

The following abbreviations are used in this manuscript:

AOAC	Association of Official Analytical Chemists
BOD	Biologica Oxygen Demand
CAGR	Compound Annual Growth Rate
CNB	Coriaria Nepalensins bark
COD	Chemical Oxygen Demand
DSC	Differential Scanning Calorimetry
EB(%)	Elongation at break (percentage)
HS	Hydrothermal Stability
IUP	International Union for Physical testing
SEM	Scanning Electron Microscope
T(%)	Tannin (percentage)
T/N	Tannin/Non-tannin
T_d_	Denaturation Temperature
TDS	Total Dissolved Solids
TFC(%)	Total Flavonoid Content (percentage)
TNBSA	2,4,6-trinitrobenzenesulphonic acid
TPC(%)	Total Phenol Content (percentage)
TS	Tensile Strength
Ts	Tear Strength
Ts	Shrinkage Temperature
UNIDO	United Nations Industrial Development Organization
VT	Vegetable Tannin
