# Supplementary material for: Could Fostering Alternative Plant Feedstocks Improve the Sustainability of Leather Manufacturing? A Critical Review
Source: Materials (Basel). 2025 Aug 11;18(16):3759. doi: 10.3390/ma18163759 (PMC12387617; doi:10.3390/ma18163759)
Supplement: Supplementary file 1 [file materials-18-03759-s001.zip › materials-3754113-supplementary.pdf]

Supporting Information

May Fostering Alternative Plant Feedstocks Improve the Sustainability of Leather Manufacturing? A Critical Review

Valentina Beghetto[1,2,3]*, Vanessa Gatto[1] and Silvia Conca [1]

[1] Crossing S.r.l., Viale della Repubblica 193/b, 31100, Treviso, Italy; silvia.conca@crossing-srl.com, vanessa.gatto@crossing-srl.com
[2] Department of Molecular Sciences and Nanosystems, University Ca' Foscari of Venice, Via Torino 155, 30172, Mestre, Italy; beghetto@unive.it
[3] Consorzio Interuniversitario per le Reattività Chimiche e La Catalisi (CIRCC), Via C. Ulpiani 27, 70126, Bari, Italy
* Correspondence: beghetto@unive.it

List of different pretanning, tanning and retanning protocols employed in papers reviewed

Table S1. Recipe for tanning of sheep pickle pelt using *Cassia singueana* and Mimosa extract [1]

Type of process	Chemicals	Temperature (°C)	Amount (%)	Duration (min)	Remark
Pickilng	Water		50		Check °Be 6–8
	Common salt		7	15	
	Formic acid (1:10)		0.3	30	Check pH 3.5–4.0
	Sulphuric acid (1:20)		0.2	60	Check pH 3.5–4.0
	Drain 50% of the float				
Tanning	Mimosa (standard)	37	25	120	Check penetration
	Cassia singueana		25	120	Check penetration
	Formic acid (1:10)		1	60	Check pH 3.8–4.2
Washing	Water	35	100	30	Drain
	Water	35	100	15	Drain
Fatliquoring	Fasfol Sc		2	60	Check exhaustion
	Lipsol J-622		1.5	60	Check exhaustion
Finishing	Leather was hanging on overhead track for overnight				

Table S2. Recipe for tanning of goat skin using *Solanum incanum* and mimosa extract [2]

Type of process	Chemicals	Temperature (°C)	Amount (%)	Duration (min)	Remark
Pickilng	Water		50		Check °Be 7–8
	Common salt		10		
	Formic acid (1:10)		0.3	30	Check pH 3.5–4.0
	Sulphuric acid (1:20)		0.2	60	Drain 50% of float
Tanning	Mimosa (control) done in three portion		25	90	Check complete penetration
	Solanum incanum in three portion		25<br>25	120<br>90	Check complete penetration
	Formic acid (1:10)	37	1	60	Check pH 3.8–4.2
Washing	Water at 35°C	35	100	30	
	Water at 35°C		100	15	Drain
Fatliquoring	Fasfol Sc		2	60	Check liquor exhaustion
	Lipsol J-622		1.5	60	Check liquor exhaustion
Finishing	Leather were horsed up overnight then dried by nailing				

Table S3. Recipe for tanning of sheep skin using *Hagenia abyssinica* and mimosa extract [3]

Type of process	Chemicals	Temperature (°C)	Amount (%)	Duration (min)	Remark
Tanning	Water	30	100		pH: 3.8-4.5 leave overnight
	Mimosa (control) done in three portion		5<br>8	120<br>120	
	H. abyssinica in three portion		8	120	
	Next day				
	Fat-liquor (Lipsol J-622)		2	60	
	Formic acid (1:10)		1	120	pH 3.8–4.2 collect water for tests
	Water	37	100	20	Wash twice separately then drain

	Fungicides		0.05	40	Drained, pile & left 2 days for aging
Post tanning:					
Wet back	Water		200	overnight	Drained
	Wetting agent		1		
Neutralization	Water		50		pH:5-6
	Sodium formate		1	60	
	Genetan neutron A2		1		
	Sodium bicarbonate		0.5	60	
Retanning	Water		50	120	Drained and washed
	H. abyssinica for exp. Mimosa for control		4		
Dyeing	Water		70		
	Leather blue BR (for both expt. and control)		1	60	
	Syntal tan 40 ACC		2		
	Syntan SO		2	overnight	Check dyeing by cutting
	Retinal MD 80		2		
Fatliquoring	Water	55	100		
	Lipsol J622		3	60	Checked liquors exhaustion
	Syntal FL 329		3		
	Formic acid (1:10)		1	45	Drained, rinsed sammed and set to dry

Table S4. Recipe for tanning of goat skin using *Pontederia crassipes* and mimosa extract [4]

Type of process	Chemicals		Temperature (°C)	Amount (%)	Duration (min)	Remark
Tanning	Water	Water	25	100	120	pH:3.8-4.3
	Mimosa	*Pontederia crassipes*		7.5x4		
	Sodium bicarbonate (1:20)	Sodium bicarbonate (1:20)		0.5		
		Ethanol		10		
	Sodium thiosulphate	Sodium thiosulphate		1		
Retanning	Water	Water	45			
	Mimosa	*Pontederia crassipes*		5x3	60x3	
	Formic acid	Formic acid		0.5	15x2	

Table S5. Recipe for tanning of goat skin using *Xylocarpus granatum* and mimosa extract [5]

Type of process	Chemicals		Temperature (°C)	Amount (%)	Duration (min)	Remark
Tanning	Water	Water	25	80	120	pH:3.8-4.0
	Pretanning agent	*X. granatum*		3		
	Sodium bicarbonate (1:20)	Sodium bicarbonate (1:20)		0.8	20x3&120	
	Fungicide	Fungicide		0.2	35	
Aging, shaving, conventional steps, and processes were done						
Retanning	Water	Water	45	150	60x3	Kept overnight
	Mimosa	*X. granatum*		6x3		

Quebracho	-		6		
Formic acid	Formic acid		0.5	15x2	
After retanning other conventional processes were done for making crust leather					

Table S6.Recipe for tanning of goat skin using *Azadirachta indica* and mimosa extract [6]

Type of process	Chemicals	Temperature (°C)	Amount (%)	Duration (min)	Remark
Tanning	Water	30°C	80		
	Azadirachta indica leaf powder/extract/mimosa		8	60	
	Magnesium oxide			20x3+240	pH: 3.8-4.2
After few days of piling, the leather is shaved (thickness 0.8 mm), and then the retanning process commences as follows					
Retanning	Water	30	100		
	EDTA		0.5		
	Acrylic resin		4	30	
	Azadirachta indica leaf powder/extract/mimosa		20	60	Penetration check
	TiO_2-based replacement syntan (anti-oxidizing agent)		6		
	Atmospheric syntan		4		
	Dispersing syntan		2		
	Lanoline oil		2		
	Surface shinning agent (prevent darkening)		1		
	Molasse		5	30	Proper drain and wash
Fatliquoring	Water	45	150		
	Synthetic oil		2	60	
	Semi-synthetic oil		2		
	Lecithin oil		1		
	Neats foot oil		1		
Top fat and rinsing were carried out followed by mechanical operations.					

Table S7. Recipe for tanning of goat skin using *Eucalyptus globulus* and *A. nilotica* extract [7]

Type of process	Chemicals	Temperature (°C)	Amount (%)	Duration (min)	Remark
De-pickle	NaCl solution Baume 6-7	25	250	240	Adjust pH 2.5-3 using formic acid
	Sodium formate to adjust pH at 2.5-3				
	Degreasing Agent				
	Sodium Carbonate				
Washing	Water	25	Plenty of water	30	Drain
Tanning	Bark extract	25	350mL	240-300	
Soaking	Bark extract	25	-	Overnight	
Tanning	Adjust pH 4 with formic acid	25	-	45	
Retanning	Fatliquor,formic acid to adjust pH at 3.5	25	30	45	Drain
Washing	Water	25	Plenty of water	20	

Table S8. Recipe for tanning of sheep skin using *Coriaria nepalensis* and valonia extract [8]

Type of process	Chemicals	Temperature (°C)	Amount (%)	Duration (min)	Remark
Recover acid	Water	25	150		

and wet	Sulfuric acid		0.8	10	pH: 3
	Sodium chloride		8	60	
Neutralizing	Sodium bicarbonate	25	0.6	180	pH: 5.5
Pretanning	*Coriaria nepalensis*/valonia	25	2	60	
Tanning	Sulfited fish oil		2		
	Coriaria nepalensis/valonia	25	5	60	
	Coriaria nepalensis/valonia	25	5	120	
	Coriaria nepalensis/valonia	25	8	180	
Acidification	Formic acid		1		pH: 3.5
Washing	Water	25	100	10	
Fatliquoring	Water		100		
	LQ-5	45	8	120	
	Formic acid		1	60	pH: 4
Stopped drum, dry					

Table S9: Recipe for tanning of skin using *Eichhornia crassipes* (10 %wt and 20%wt), combination of *Eichhornia crassipes* and quebracho (5 %wt+5 %wt) and quebracho (10 %wt) [9]

Type of process	Chemicals	Temperature (°C)	Amount (%)	Duration (min)	Remark
Tanning	Veg Tan x		12	180	
	Quebracho		12		
	Veg Tan x		5		
	Quebracho		5		
	Fish oil		2	300	Penetration
	Water		100	20	
	Taningal BL		0.8	60	pH
Wet back	Water	40	200		
	Wetting agent		0.5	40	
Neutralization	Water	30	100		
	Formic acid		0.2	10	
	LSF 100		2	30	
	Sodium formate		2	30	
	Sodium bicarbonate		1.5	60	Check pH: 4.8-5
Retanning/deying and fatliquoring	Water	40	50		
	Acrylic resin		2	30	
	Retanal MD 80		4	30	
	LSF 100		4	60	
	Mimosa/ *Eichhornia crassipes*		4		
	Black dye		3	60	
	Water	60	100		
	Lipsol J622		4	60	
	Neopristol SW		1	60	
	Formic acid		2	40	Check pH: 3.8-4.2

Table S10. Recipe for tanning of goat skin using *Cassia fistula* extract [10]

Type of process	Chemicals	Temperature (°C)	Amount (%)	Duration (min)	Remark
Tanning	Water	25	80	120	pH: 3.9-4
	C. fistula		4		
	Sodium bicarbonate		0.8	20x3	
	Fungicide		0.1	35	
Aging, shaving, and traditional steps were carried out					
Retanning	Water	45	150	60x3	Kept for overnight
	C. fistula		6x3		
	Syntan		6x3		

	Synthetic fat		1x3		
	Formic acid		0.5		

Table S11. Recipe for tanning of goat skin using *Rumex abyssinicus* and mimosa extract [11]

Type of process	Chemicals	Temperature (°C)	Amount (%)	Duration (min)	Remark
Adjustment of pH	Water		100		
	Sodium bicarbonate		0.5	2x15	Adjust pH 4, 5 and 6
Tanning	Phenolic syntan		2	30	
	Rumex abyssinicus/ mimosa (control)		5-30 (*Rumex abyssinicus*) 15 (mimosa)	120	
Fixing	Formic acid		0.25	3x10+30	Check pH 3.5
Washing	Water		300	10	
Neutralization	Water		200		
	Sodium bicarbonate		0.75	3x15	pH 5-5.5
	Water		100		
Post tanning	Synthetic tanning agents		6	40	
	Dye		3	40	
	Fatliquor		7	30	
Fixing	Formic acid		0.25	3x10+30	pH 3.5
Washing	Water		300	10	

Table S12. Recipe for tanning of sheep skin using *Osyris lanceolata* extract [12]

Type of process	Chemicals	Temperature (°C)	Amount (%)	Duration (min)	Remark
De-pickilng	Water	30	80		
	Sodium chloride		8	10	Beume (B/O) was checked, it was between 6 and 8
	RECURCAD PHE (aldehyde type)	30	2	60	
	Sodium bicarbonate		1	20	
	Sodium bicarbonate		0.5	20	
	RecurCAD NT		2	30	Check pH: 3-4.5, drain
Tanning	Water		100		
	Osyris lanceolata powder solution	45	10		
	Bastamol K		5	60	
	Osyris lanceolata powder solution		20		
	Basyntan D, (retanning agents)		3	60	
	Water	55	100		
	Brown dye		3	30	
	Coriamin SA/N (Cationic fatliquor)		3	120	Check pH: 5.7
	Catalix® LX liq (lightfast synthetic fat-liquor)		3		
	Polymer RSC		3		
Fixation	Water		200	60	
	Oxalic		0.1		
Drain	Water	50	150	15	

Rinsing	Water	30	300	20

References

1. Teklemedhin, T.B.; Gebretsadik, T.T.; Gebrehiwet, T.B.; Gebrekidan, G.A.; Edris, M.; Teklegiorgis, N.T.; Hagos, K.B. Vegetable Tannins as Chrome-Free Leather Tanning. *Adv. Mater. Sci. Eng.* **2023**, *2023*, 1–11, doi:10.1155/2023/6220778.
2. Seda Badessa, T.; Hailemariam, M.T.; Ahmed, S.M. Greener Approach for Goat Skin Tanning. *Cogent Eng.* **2022**, *9*, 2018959, doi:10.1080/23311916.2021.2018959.
3. Unango, F.J.; Duraisamy, R.; Ramasamy, K.M.; Birhanu, T. Characteristics and Tanning Potential of *Hagenia Abyssinica* Tannin Extracts and Its Possible Use in Clean Leather Production. *Cogent Eng.* **2021**, *8*, 1993520, doi:10.1080/23311916.2021.1993520.
4. Mustafa, M.A.; Noyon, M.A.R.; Uddin, M.E.; Islam, R. Sustainable Leather Tanning with Pontederia Crassipes Tannin: A Promising Eco-Friendly Alternative. *Clean. Eng. Technol.* **2024**, *18*, 100717, doi:10.1016/j.clet.2023.100717.
5. Das, R.K.; Mizan, A.; Zohra, F.T.; Ahmed, S.; Ahmed, K.S.; Hossain, H. Extraction of a Novel Tanning Agent from Indigenous Plant Bark and Its Application in Leather Processing. *J. Leather Sci. Eng.* **2022**, *4*, 18, doi:10.1186/s42825-022-00092-5.
6. Shakil, S.R.; Zenith, F.T.J.; Khan, M.R.; Tonay, W.R. Application and Valorization of Novel Indigenous Azadirachta Indica Leaf in Leather Processing. *Heliyon* **2024**, *10*, e36270, doi:10.1016/j.heliyon.2024.e36270.
7. Khan, S.R.; Khan, S.M.; Khan, R.U. Eco-Friendly Valorization and Utilization of Plant Waste as a Source of Tannin for Leather Tanning. *Sustainability* **2023**, *15*, 3884, doi:10.3390/su15053884.
8. Guo, L.; Qiang, T.; Ma, Y.; Wang, K.; Du, K. Optimisation of Tannin Extraction from Coriaria Nepalensis Bark as a Renewable Resource for Use in Tanning. *Ind. Crops Prod.* **2020**, *149*, 112360, doi:10.1016/j.indcrop.2020.112360.
9. Ahmed, F.E.; Gelebo, G.G.; Gebre, B.M. Potential of Water Hyacinth Leaves Extract as a Leather Tanning Agent. *J. Am. Leather Chem. Assoc.* **2022**, *117*, 391–399, doi:10.34314/jalca.v117i9.6169.
10. Oaishi, R.T.; Ahmed, S.; Tuj-Zohra, F.-; Rahman, A.; Abid, N.M. Facile Extraction and Prospective Application of Indigenous *Cassia Fistula* Tannin in Sustainable Leather Manufacture. *J. Dispers. Sci. Technol.* **2024**, *45*, 2134–2145, doi:10.1080/01932691.2023.2247073.
11. Mohammed, S.A.; Naisini, A.; Madhan, B.; Demessie, B.A. *Rumex Abyssinicus* (Mekmeko): A Newer Alternative for Leather Manufacture. *Environ. Prog. Sustain. Energy* **2020**, *39*, e13453, doi:10.1002/ep.13453.
12. Teshome, Z.; Agazhi, T.; Gashew, T.; Solomon, B.; Belay, T.; Baye, B.; Temach, E. Extraction and Optimization of Tanning Material from Osyris Lanceolata Barks: Cleaner Leather Tanning Processing. *J. Chem.* **2023**, *2023*, 1–12, doi:10.1155/2023/6610247.
